# N6-Methyladenosine Regulators Promote Malignant Progression of Gastric Adenocarcinoma

**DOI:** 10.3389/fonc.2021.726018

**Published:** 2022-03-01

**Authors:** Yibin Zhao, Xiao Yan, Yu Wang, Juan Zhou, Yang Yu

**Affiliations:** ^1^ Department of Anus & Intestine Surgery, Ningbo Medical Center LiHuiLi Hospital, Ningbo, China; ^2^ Department of Hematology, Ningbo First Hospital, Ningbo, China; ^3^ Department of Medical Oncology, Xuzhou Central Hospital, Xuzhou, China

**Keywords:** N6-methyladenosine (m6A), pan-cancer, gastric adenocarcinoma, risk score, Cox regression, TCGA, FTO

## Abstract

N6-methyladenosine (m6A) RNA methylation is dynamically and reversibly regulated by methyltransferases, binding proteins, and demethylases. The restoration of m6A to adenosine could result in demethylation modifications. Abnormalities in m6A epigenetic modifications in cancer are of increasing interest in recent years. According to the progression and prognostic performance of m6A epigenetic modifications in gastric adenocarcinoma (STAD), this study comprehensively analyzed the m6A modification patterns of gastric adenocarcinoma specimens in The Cancer Genome Atlas (TCGA) database based on 20 m6A regulators. Here, we found that 20 m6A RNA methylation regulators were high-expressed in gastric adenocarcinoma. m6A RNA methylation regulators were closely associated with pT staging of gastric cancer. Based on such findings, we developed a prognostic model using four m6A RNA methylation regulators (IGF2BP1, RBM15, FTO, ALKBH5), and the FTO was confirmed as an independent prognostic marker.

## Introduction

The incidence of digestive diseases is increasing with the changes of modern diet (1). Stomach adenocarcinoma (STAD) is a common malignant digestive system tumor (2). Despite some progresses in STAD treatment in recent years, STAD remains the second leading cause of cancer death-related (3). Statistics have shown that more than 700,000 deaths from STAD have occurred annually all over the world (4). Such a high mortality rate is attributed to the specific biological features of the disease, such as a lack of efficient clinical diagnostic indicators, unclear clinical presentation, and high invasion and metastasis degrees (5), which also point to the necessity of identifying effective biomarkers and their potential molecular mechanisms in STAD (6). The completion of the Human Genome Project (HGP) and parallel development of next-generation sequencing (NGS) have been accompanied with increasing technological advances (7). Many major oncology research programs have been launched, the most famous of which are The Cancer Genome Atlas (TCGA) (8) and Genotype-Tissue Expression (GTEx) (9). The aim of these two programs is to map the genome of human tumors through large-scale high-throughput genome sequencing and microarray technology to find new solutions for cancer treatment through exploring tumor development and potential molecular mechanisms.

Epigenetics, which becomes a popular research area in recent years, is defined as DNA sequence invariance but genetic alteration of gene expression (10). Earlier studies found that epigenetic studies focused on DNA and histone modifications (11). Some scholars believed that mRNAs only play a role in information transfer (12). However, with the rapid development of high-throughput sequencing technology, studies have increasingly found that mRNAs, for example, N6-methyladenosine (m^6^A), N1-methyladenosine (m^1^A) and pseudo uridine methylation (13), undergo various modifications during 5'-capping and 3'-tailing 4-6 under exon splicing (14). These above modifications can exert certain effects on the splicing, nucleation, stabilization, and translation of mRNA, which further affects the mRNA metabolic process and regulates gene expression (15). Up to now, 171 RNA modifications have been identified (16), and m6A as an important modifier is the most abundant in a variety of eukaryotic mRNAs and long-stranded noncoding RNAs (LncRNAs) (13). RNA m6A can be imprinted by methyltransferases, preferentially recognized and delivered by reader proteins, and cleared by RNA methylases, suggesting that m6A methylation epigenetically mediates the expression of a large number of genes, thereby playing multiple roles in regulating biological processes (17).

Previous studies have shown that multiple proteins are involved in the regulation of m6A. For example, METTL3, METTL14 can act as compilers (18); YTHDF1, YTHDF2, YTHDF3 can act as readers (13); FTO, ALKBH5 can act as erasers (19). Recent studies have reported the association of m6A regulators with cancer. The diagnostic, progressive, and prognostic performance of m6A-methylated RNA regulators in lung adenocarcinoma has been confirmed (20). Moreover, m6A modification-mediated CBX8 induction could regulate the stemness and chemosensitivity of colon cancer through upregulation of LGR5 (21).

Only 13 m6A RNAs had been analyzed in earlier studies, currently new research has identified 7 m6A RNAs. In the present study, we analyzed the differential expression of 20 m6A RNA regulators in STAD through TCGA and GTEx databases, and found that the expression of m6A RNA methylation regulator FTO played an important role in the progression of STAD and was considered as an effective prognostic factor.

## Materials and Methods

### Data Collection

Data from 375 gastric adenocarcinomas (STAD) tumor samples were downloaded from The Cancer Genome Atlas (TCGA, https://portal.gdc.cancer.gov/) website and Genotype-Tissue Expression (GTEx, https://gtexportal.org/) website to acquire 361 cases of normal tissues.

### Selection and Processing of Study Genes

The collected data were normalized and 361 normal lung tissues from GTEx were imported into the STAD TCGA data set to increase the number of normal groups. Differential analysis on 20 m6A RNA methylation genes from normal and tumor tissues was performed using the Wilcox test. 20 m6A RNA methylation genes, namely, HNRNPA2B1, VIRMA, METTL3, WTAPR, HNRNPA2B1, METTL14, BM15, RBM15B, ZC3H13, YTHDC1, YTHDF3, YTHDF1, YTHDF2, HNRNPC, IGF2BP1, IGF2BP2, IGF2BP3, RBMX, FTO, ALKBH5, were introduced in the current study. Correlations between genes were analyzed with the corrlot package.

### Gene Enrichment Analysis

Samples were divided into high- and low- expression groups based on median FTO expression value. FTO biological functions were analyzed using gene enrichment analysis (GSEA) (http://software.broadinstitute.org/gsea/index.jsp). The Kyoto Encyclopedia of Genes and Genomes (KEGG) pathway, Gene Ontology (GO), and Hallmarkgene signature gene collections were used for analysis, and annotation was performed using the "clusterProfiler" package.

### Hierarchical Clustering of m6A RNA Methylation Genes and Correlation Analysis

The Consensusclusterplus package was used for consistency analysis. The maximum number of clusters was 6. 80% of the total samples, which were extracted by 100 repetitions. The clustering heat map was plotted by pheatmap package. Kaplan-Meier analysis and corresponding analysis were then applied to analyze the differences between clusters.

### LASSO Prognostic Modeling

Overlapping DEGs and DMGs were filter by LASSO regression pipeline to narrow down the target genes. Genes associated with survival were screened by univariate Cox analysis, and risk characteristics for the Cox regression model were analyzed using the glmnet package and the survivor package with the following risk equation:


$$\text{Risk scores}=\Sigma_{i}^{\text{n}}X\text{i}\times Y\text{i }(\text{X}:\text{coefficient of each gene},\text{ Y}:\text{expression of each gene})$$

Based on the median score, STAD patients in the TCGA database were divided into two subgroups low-risk and high-risk groups. Kaplan-Meier survival curve analysis was used to compare the OS times of the two groups, and the prediction of the genetic markers was evaluated by time-related ROC.

### Univariate and Multivariate Cox Regression Analyses

Cox regression analysis was performed using the Survival package, and forestplots were generated with the forestplot package to show P values, HRs, and 95% CIs for each variable. Based on the results of multivariate Cox proportional risk analysis, column line plots were developed using the rms package to predict 1-, 3-, and 5-year survival rates. Column line plots provided a graphical representation of these factors, allowing the prognostic risk of individual patients to be calculated by the points associated with each risk factor.

## Results

### Differential Expression of m6A RNA Methylation Regulators in STAD

**Figure 1:**
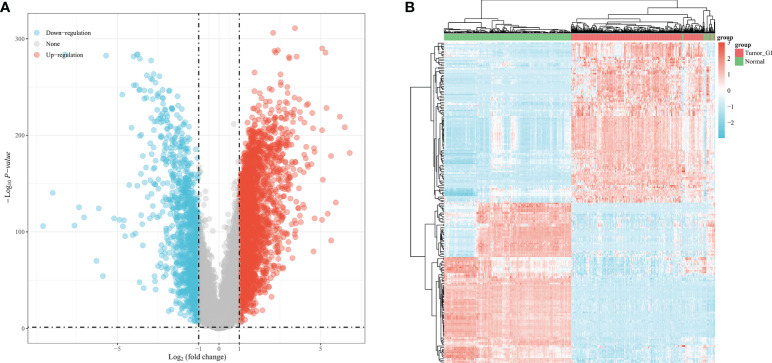
Expression levels of m6A RNA methylation regulators in TCGA database. **(A)** Expression levels of 20 m6A RNA methylation regulators in 375 STAD tissues and 32 normal tissues of the TCGA database. **(B)** Pearson's test for the correlation between the expression levels of 20 m6A RNA methylation regulators.

To further understand the important biological functions of m6A RNA methylation regulators in tumorigenesis and development. We compared the expression levels of 20 m6A RNA methylation regulators in 375 STAD tissues and 32 normal tissues from the TCGA database. The results showed that compared to normal tissues, in STAD patients the expression of HNRNPA2B1, VIRMA, METTL3, WTAPR, HNRNPA2B1, METTL14, BM15, RBM15B, ZC3H13, YTHDC1, YTHDF3, YTHDF1, YTHDF2, HNRNPC, IGF2BP1 IGF2BP2, IGF2BP3, RBMX, FTO, and ALKBH5 was significantly up-regulated (**Figure 1A**). In addition, we analyzed the correlation between 20 m6A RNA methylation regulators through conducting Pearson's test, and the analysis revealed a positive correlation between the 20 m6A RNA methylation regulators, with VIRMA and YTHDF3 showing the most significant correlation (**Figure 1B**). This suggested when VIRMA was up-regulated, the YTHDF3F gene was most likely to be upregulated.

### Consensus Clustering of m6A RNA Methylation Regulators to Analyze the Characteristics and Survival of STAD Patients

**Figure 2:**
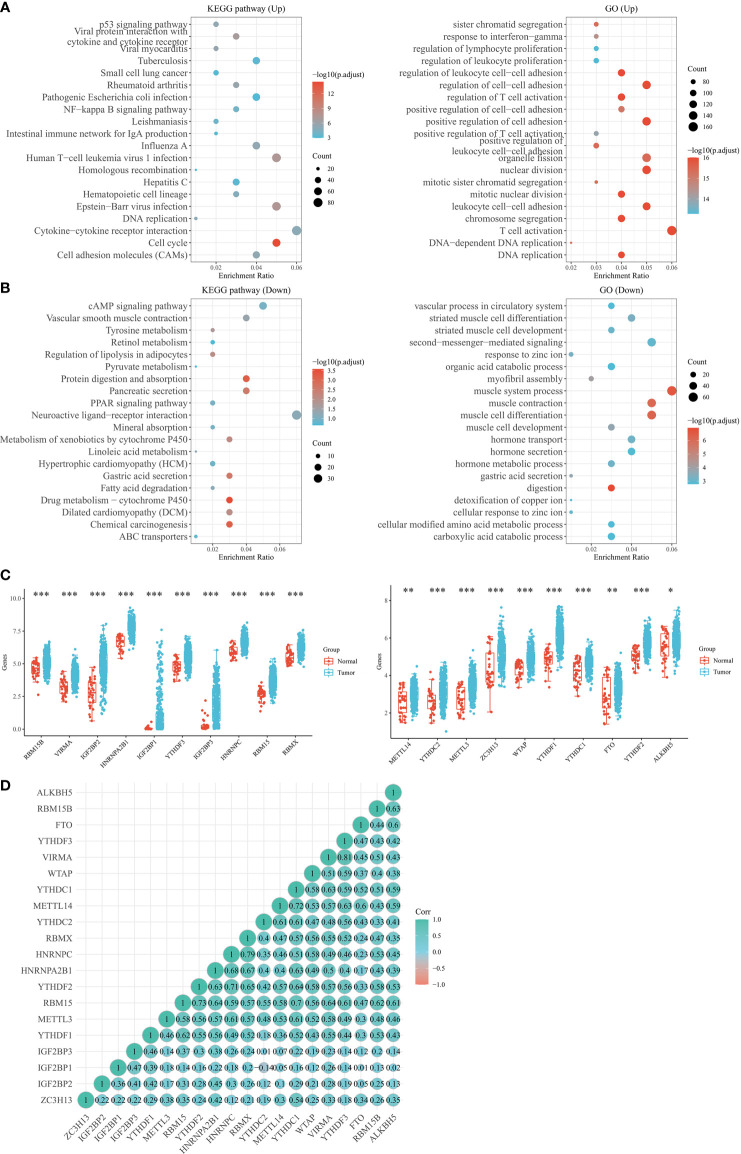
TCGA database using 20 m6A RNA methylation-associated genes to divide the samples into 3 subgroups. **(A)** The STAD cohort was separated into three clusters when k = 3. **(B)** CDF curves for k=2~6. **(C)** Relative amount of change in area under the CDF curve for consensus clustering when k=2~6. **(D)** The distribution of each sample when k is in the range of 2 ~ 6. CDF, cumulative distribution function. *p < 0.05; **P < 0.01; ***P < 0.001.

**Figure 3:**
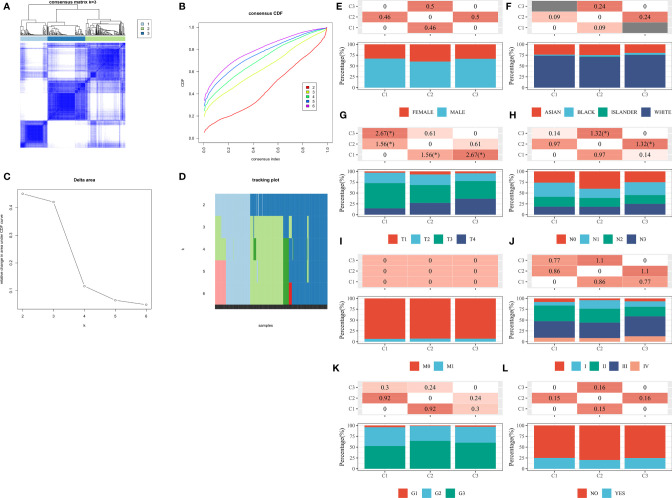
Consensus clustering of m6A RNA methylation regulators to analyze the characteristics and survival of STAD patients. **(A)** The STAD cohort was separated into three clusters when k = 3. **(B)** CDF curves for k=2~6. **(C)** Relative amount of change in area under the CDF curve for consensus clustering when k=2~6. **(D)** The distribution of each sample when k is in the range of 2 ~ 6. **(E)** Comparison of gender differences among the 3 subgroups. **(F)** Comparison of race differences among the 3 subgroups. **(G)** Comparison of T-stage differences among the 3 subgroups. **(H)** Comparison of N-stage differences among the 3 subgroups. **(I)** Comparison of M-stage differences among the 3 subgroups. **(J)** Comparison of clinical staging differences among the 3 subgroups. **(K)** Comparison of differences in differentiation in 3 subgroups. **(L)** Comparison of differences in lymphatic metastasis among the 3 subgroups. Note: CDF, cumulative distribution function. *P < 0.05, **P < 0.01, ***P < 0.001.

To better understand the relationship between m6A RNA methylation regulators and clinical characteristics of STAD patients, we extracted 32 normal samples from the TCGA database and employed the ConsensusClusterPlus package to subclassify 343 STAD samples. Based on the similarity shown by the expression levels of m6A regulators and the ratio of fuzzy clustering metrics, the optimal clustering stability was defined from k = 2 to 6 when k = 3 (**Figure 2**). Subsequently, 375 STAD patients were divided into 3 subgroups, namely, cluster1 (n = 97), cluster2 (n = 143) and cluster3 (n = 135), according to the expression levels of m6A regulators. Next, we analyzed the clinical characteristics and survival of the 3 subgroups. First, we examined the differences between gender, race, pT stage, pN stage, pM stage, pTNM stage, and Grade in the 3 subgroups of patients with sub STAD. After comparing cluster1, cluster2, and cluster3, we found that there was a difference in pT staging between them as well as a difference in pN staging between cluster2 and cluster3, while other clinical data were not significant (**Figure 3**).

### LASSO Prognostic Model Construction

**Figure 4:**
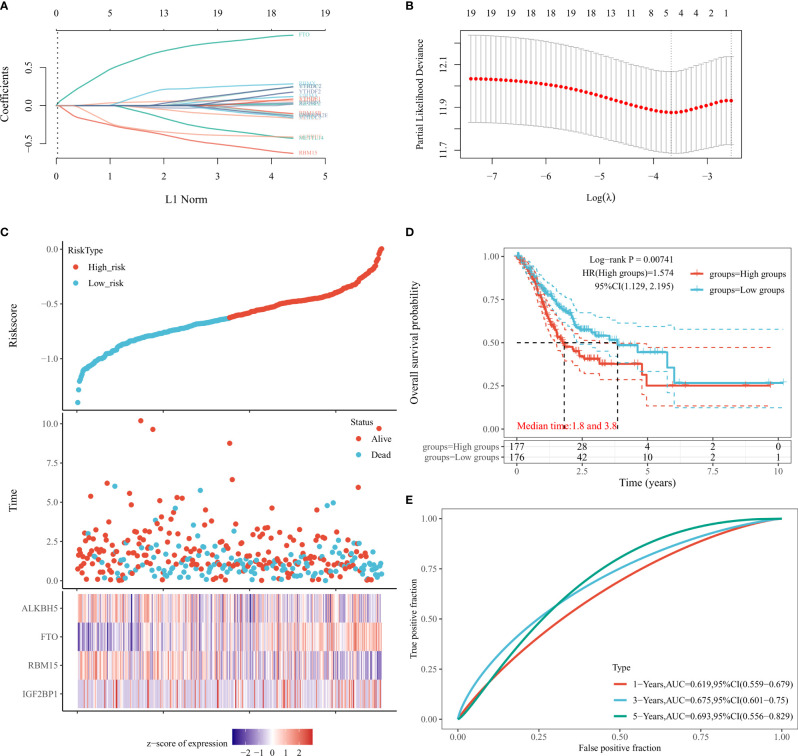
Relationship between m6A RNA methylation regulators and prognosis of STAD patients by constructing LASSO models in TCGA database. **(A, B)** Relationship between the solution path of LASSO model and cross-validation mean square error (CV MSE) and model size. **(C)** Risk assessment subgroups of STAD patients and the expression levels of IGF2BP1, RBM15, FTO, and ALKBH5 within the subgroups. **(D)** Kaplan-Meier curves of STAD patients in the high-risk and low-risk groups according to risk scores. **(E)** ROC curves to validate the LASSO model for 1-, 3-, and 5-year prediction efficiency.

The LASSO Cox regression model was applied to construct prognostic features to analyze the expression levels of m6A RNA methylation regulators (**Figures 4A, B**). According to the minimal criterion, four regulators, IGF2BP1, RBM15, FTO, and ALKBH5, were selected to construct the prognostic model. The risk score for each STAD patient was calculated with the following formula: risk score = (0.0355)*IGF2BP1 + (-0.2487)*RBM15 + (0.4701)*FTO + (-0.2246)*ALKBH5. Based on the median risk score, the STAD cohort was divided into low-risk group and high-risk group (**Figure 4C**). Kaplan-Meier survival analysis demonstrated that patients in the high-risk group had significantly higher OS than those in the low-risk group (P=0.007, **Figure 4D**). In addition, the sensitivity and specificity of this model for predicting patients' OS period were verified by ROC curves. Here, the risk model showed a better accuracy in predicting survival at 1 year (AUC=0.743), 3 years (AUC=0.743), and 5 years (AUC=0.874) after surgery (**Figure 4E**). This suggested that the model was accurate in predicting the prognostic survival of STAD patients.

### Univariate and Multivariate Cox Regression Analyses on m6A RNA Methylation Regulators

**Figure 5:**
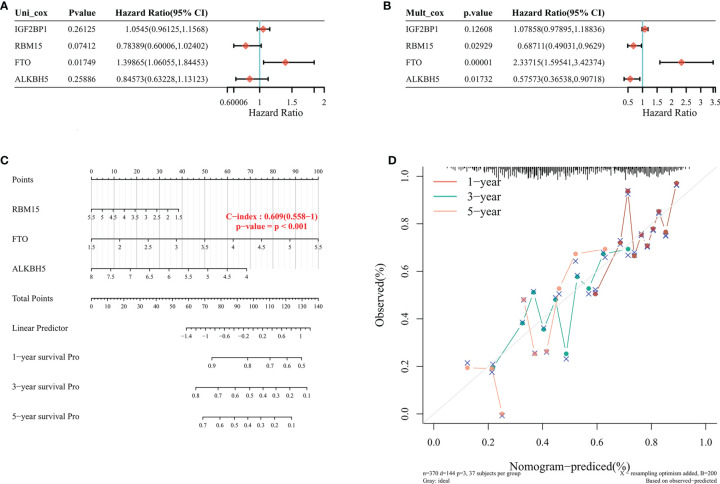
Nomogram plot construction in TCGA database. **(A, B)** Univariate and multifactorial cox analysis of p-value, risk factor HR, and confidence interval for 4-m6A RNA methylation regulator expression and clinical characteristics. **(C)** Columnar plots to predict 1-year, 3-year, and 5-year overall survival of STAD patients **(D)** Calibration curves of the overall survival line plot model in the discovery group. The diagonal dashed line indicates the ideal column line plot, the blue, red, and orange lines indicate the observed 1-year, 3-year, and 5-year column line plots.

We analyzed whether IGF2BP1, RBM15, FTO, and ALKBH5 genes were independent prognostic factors for STAD. Cox regression analysis, and univariate and multivariate Cox regression analysis revealed that FTO (HR: 2.337, 95% CI (1.595-3.423), P<0.001) may be an independent prognostic factor for STAD (**Figures 5A, B**). To develop a clinically applicable method predictive of patients’ survival, we used Nomogram plots to construct a prediction model, and Nomogram plots were developed to predict 1-, 3-, and 5-year OS rates using the Cox regression algorithm (**Figure 5C**). It was found that the calibration plots of 1, 3, and 5-year OS rates were all highly predictive when compared to the ideal model in the whole cohort (**Figure 5D**).

### The Biological Significance of FTO in Gastric Cancer

In the above study, FTO was identified as an independent prognostic factor for STAD. To further understand the significance of FTO in STAD, we performed GSEA analysis and divided the patients into high- and low-expression groups based on the expression of FTO in STAD to observe the signaling enrichment of GO, KEGG, and markers in both groups. The 10 most enriched signaling pathways were ranked according to NES scores, and we selected the 10 most enriched signaling pathways for presentation (**Tables 1**–**3**).

**Table 1:** GO enrichment analysis (Top 10).

NAME	ES	NES	NOM p-val	FDR q-val	FWER p-val
GO RESPIRATORY CHAIN COMPLEX IV	-0.663	-1.776	0.029	1	0.864
GO OXIDOREDUCTASE ACTIVITY ACTING ON A HEME GROUP OF DONORS	-0.597	-1.764	0.033	1	0.876
GO DYNACTIN COMPLEX	-0.594	-1.764	0.012	1	0.878
GO STRUCTURAL CONSTITUENT OF MUSCLE	-0.574	-1.722	0.023	1	0.916
GO SERINE TYPE ENDOPEPTIDASE INHIBITOR ACTIVITY	-0.491	-1.714	0.023	1	0.923
GO CARDIAC RIGHT VENTRICLE MORPHOGENESIS	-0.628	-1.700	0.020	1	0.933
GO ATP SYNTHESIS COUPLED PROTON TRANSPORT	-0.624	-1.694	0.059	1	0.937
GO TERPENOID BIOSYNTHETIC PROCESS	-0.617	-1.682	0.014	1	0.953
GO INNATE IMMUNE RESPONSE IN MUCOSA	-0.643	-1.680	0.035	1	0.953
GO LOW DENSITY LIPOPROTEIN PARTICLE REMODELING	-0.637	-1.666	0.021	1	0.961

**Table 2:** KEGG enrichment analysis (Top 10).

NAME	ES	NES	NOM p-val	FDR q-val	FWER p-val
KEGG CARDIAC MUSCLE CONTRACTION	-0.426	-1.769	0.023	0.578	0.415
KEGG STEROID BIOSYNTHESIS	-0.665	-1.521	0.078	1.000	0.811
KEGG SYSTEMIC LUPUS ERYTHEMATOSUS	-0.469	-1.489	0.092	1.000	0.846
KEGG OXIDATIVE PHOSPHORYLATION	-0.412	-1.465	0.202	0.841	0.873
KEGG PARKINSONS DISEASE	-0.403	-1.447	0.188	0.729	0.893
KEGG RIBOSOME	-0.559	-1.409	0.170	0.713	0.922
KEGG PHENYLALANINE METABOLISM	-0.425	-1.325	0.136	0.843	0.960
KEGG ARACHIDONIC ACID METABOLISM	-0.367	-1.237	0.194	0.997	0.978
KEGG STEROID HORMONE BIOSYNTHESIS	-0.383	-1.208	0.246	0.972	0.983
KEGG COMPLEMENT AND COAGULATION CASCADES	-0.399	-1.207	0.267	0.878	0.983

**Table 3:** HALLMARK enrichment analysis (Top 10).

NAME	ES	NES	NOM p-val	FDR q-val	FWER p-val
HALLMARK PANCREAS BETA CELLS	-0.440	-1.353	0.164	1.000	0.698
HALLMARK COAGULATION	-0.376	-1.292	0.199	1.000	0.754
HALLMARK MYOGENESIS	-0.374	-1.256	0.260	0.821	0.787
HALLMARK EPITHELIAL MESENCHYMAL TRANSITION	-0.443	-1.201	0.336	0.729	0.829
HALLMARK CHOLESTEROL HOMEOSTASIS	-0.303	-0.983	0.466	1.000	0.937
HALLMARK ANGIOGENESIS	-0.329	-0.918	0.530	1.000	0.959
HALLMARK OXIDATIVE PHOSPHORYLATION	-0.226	-0.781	0.616	1.000	0.984
HALLMARK KRAS SIGNALING DN	-0.191	-0.757	0.861	1.000	0.985
HALLMARK REACTIVE OXYGEN SPECIES PATHWAY	-0.198	-0.698	0.796	1.000	0.989
HALLMARK HYPOXIA	-0.189	-0.683	0.816	0.969	0.990

### FTO Pan-Cancer Analysis

**Figure 6:**
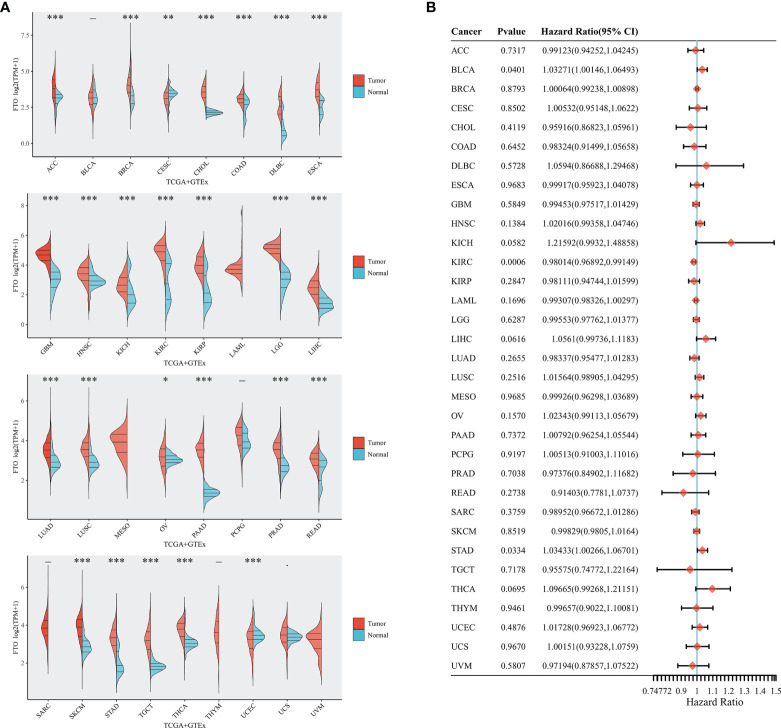
Expression of FTO in the pan-cancer and prognosis. **(A)** Expression level of FTO in pan-cancer in TCGA+GTEx database. **(B)** Prognosis of FTO expression level in pan-cancer in TCGA database. The tumor abbreviations are annotated in **Table S1**. *p < 0.05; **P < 0.01; ***P < 0.001.

We combined TCGA and GTEx databases to further compare the expression of FTO in different cancers, and found that FTO was expressed in 25 tumors, including in ACC, BRCA, CHOL, COAD, READ, DLBC, ESCA, GBM, HNSC, KICH, KIRC, KIRP, LAML, LGG, LIHC, LUAD, LUSC, MESO, OV, PAAD, PCPG, PRAD, READ, SKCM, STAD, STES, TGCT, THCA, UCEC, and showed a low expression in CESC (**Figure 6A**). In addition, the relationship between FTO expression and pan-cancer survival was analyzed based on TCGA database data. After plotting forest plots, we found that FTO expression was correlated with BLCA, KIRC, and STAD (**Figure 6B**), suggesting that FTO may have certain potential diagnostic or prognostic significance also in other tumors.

## Discussion

Gastrointestinal tract tumors are currently one of the most common malignancies in clinical practice. STAD is the fifth most common malignancy in the world and a serious threat to human health (22). At present, the pathogenetic factors of STAD were not clear. However, scholars have found that environmental factors, daily diet, H. pylori infection, and genetic inheritance can all lead to development of STAD (23, 24). Currently, the preferred treatment option for STAD is surgery combined with adjuvant chemotherapy, radiotherapy, targeted therapy, and immunosuppressive agents (25). Although the mortality rate of STAD is showing a decreasing trend, the 5-year survival of patients with advanced STAD is still below 30% (5). Targeted drug therapy has a positive effect on prolonging patient's survival time, long-term use of drug will result in drug resistance (26), which has now become one of urgent problems to be resolved in clinical practice. For this reason, further investigation of the molecular mechanisms of STAD pathogenesis and search of new therapeutic targets should be addressed.

DNA methylation is currently one of the most important mechanisms in tumor studies (27). m6A methylation is by far the most common form of mRNA modification and plays an important role in tumor development through post-transcriptional regulation (28). Recent studies indicated that dysregulation of m6A methylation regulatory proteins can induce downstream RNA metabolism disorders (29). Guo et al. (30) found that RNA demethylase ALKBH5 inhibits pancreatic cancer progression through post-transcriptional activation of PER1 in m6A-YTHDF2-dependent manner, and Zhang et al. (31) showed that YTHDF2 supresses pancreatic cancer progression through m6A RNA methylation regulating OCT4 expression to promote hepatocellular carcinoma stem cell phenotype and tumor metastasis. However, not all m6A modifiers of m6A modification are in a suppressed statues in tumors. Shen et al. (32) found that ALKBH5 expression deficiency is a poor prognostic indicator to acute myeloid leukemia. Zhang et al. demonstrated that ALKBH5 expression is up-regulated in gliomas, and this can promote glioma development and progression through up-regulating ALKBH5-mediated FOXM1 (33). These findings indicated that the regulation of m6A methylation modification levels is highly complex. Moreover, current studies on m6A methylation modifiers were mainly focused on oncogenic pathways and have not explored m6A regulators in depth.

In the current study, through comprehensive analyses, we found that m6A RNA methylation regulators were involved in the development of STAD, and that m6A RNA methylation regulators were associated with STAD pathological features. In addition, for a better analysis of m6A RNA methylation regulators, we applied consensus clustering, a more efficient clustering method allowing a better assessment of cluster stability through performing multiple iterations of the clustering method. In this study, we divided the STAD samples into 3 subgroups (cluster1, cluster2, and cluster3) by consensus clustering. The analytical results showed that subgroup levels were correlated with pT, and pN staging. Finally, through building a prognostic model, we developed a prognostic genetic marker (FTO), which could classify the OS of STAD patients into low- and high-risk subgroups. Cox regression analysis showed that FTO notation can be used as a potential independent prognostic marker and a predictor of clinicopathological parameters.

FTO, a nuclear protein of the AlkB-related superfamily of non-heme iron and 2-oxoglutarate-dependent oxygenases, is an important demethylase (34). Existing studies showed a strong correlation between FTO in humans and body mass index (35), obesity risk (36), and type 2 diabetes (37). FTO consumption could increase total m6A levels in polyadenylated RNAs (38). m6A is oxidized by FTO and N6-hydroxymethyladenosine (Hm6A), and N6-formyladenosine (F6A) are produced during the oxidation process (39). The potential functions of the intermediates produced during this oxidation process remains unclear. To further clarify the mechanisms of FTO in STAD, we found that FTO was involved in cellular oxidation, epithelial-mesenchymal transition, and other functions by conducting single gene enrichment analysis. Earlier studies showed that FTO is not only expressed in STAD, but also in acute myeloid leukemia (40), cervical squamous carcinoma (41), and glioblastoma (42), indicating that FTO may be involved in the development of other tumors. To further determine the expression of FTO in other tumors, the expression of FTO in 32 tumors was analyzed based on the TCGA database and the GTEx database. Here, we found that FTO was high-expressed in a majority of tumors and was considered as a risk factor for STAD patients in prognostic analysis. This indicated that FTO could be expected to be an independent prognostic and potential therapeutic target for STAD. The above study showed the significance of FTO in STAD, which provided new evidence for the pathogenesis and potential targets of tumors and new ideas for tumor gene-targeting therapy. However, this study still had some limitations, as this study did not carry out experimental validation, and the conclusions obtained through bioinformatics analysis should be further confirmed *in vivo* and *in vitro*. Therefore, in later studies, we plan to conduct clinical trials and basic experiments to verify the the findings of this study.

In conclusion, our results systematically demonstrated the expression, potential function, and prognostic value of the m6A RNA methylation regulator FTO in STAD, contributing to tumor gene-targeting therapy and clinical prognosis study.

## Data Availability Statement

The original contributions presented in the study are included in the article/**Supplementary Material**. Further inquiries can be directed to the corresponding author.

## Author Contributions

All authors listed have made a substantial, direct, and intellectual contribution to the work and approved it for publication.

## Funding

This work was supported by Natural Science of Ningbo Foundation (2015A610223), Key Research And Development Project For Science And Technology Innovation of Xuzhou City (KC20094), Xuzhou key clinical physician training program (2020) and Ningbo Clinical Medicine Research Center Project (grant no. 2019A21003)

## Conflict of Interest

The authors declare that the research was conducted in the absence of any commercial or financial relationships that could be construed as a potential conflict of interest.

## Publisher’s Note

All claims expressed in this article are solely those of the authors and do not necessarily represent those of their affiliated organizations, or those of the publisher, the editors and the reviewers. Any product that may be evaluated in this article, or claim that may be made by its manufacturer, is not guaranteed or endorsed by the publisher.
