# Supplementary material for: N6-Methyladenosine Regulators Promote Malignant Progression of Gastric Adenocarcinoma
Source: Front Oncol. 2022 Mar 1;11:726018. doi: 10.3389/fonc.2021.726018 (PMC8921461; doi:10.3389/fonc.2021.726018)
Supplement: Supplementary file 1 [file Table_1.docx]

**Table S1** TCGA Abbreviations

| Tumor | Abbreviations |
| --- | --- |
| Adrenocortical carcinoma | ACC |
| Bladder Urothelial Carcinoma | BLCA |
| Breast invasive carcinoma | BRCA |
| Cervical squamous cell carcinoma and endocervical adenocarcinoma | CESC |
| Cholangiocarcinoma | CHOL |
| Colon adenocarcinoma | COAD |
| Lymphoid Neoplasm Diffuse Large B-cell Lymphoma | DLBC |
| Esophageal carcinoma | ESCA |
| Glioblastoma multiforme | GBM |
| Head and Neck squamous cell carcinoma | HNSC |
| Kidney Chromophobe | KICH |
| Kidney renal clear cell carcinoma | KIRC |
| Kidney renal papillary cell carcinoma | KIRP |
| Acute Myeloid Leukemia | LAML |
| Low grade glioma | LGG |
| Liver hepatocellular carcinoma | LIHC |
| Lung adenocarcinoma | LUAD |
| Lung squamous cell carcinoma | LUSC |
| Mesothelioma | MESO |
| Ovarian serous cystadenocarcinoma | OV |
| Pancreatic adenocarcinoma | PAAD |
| Pheochromocytoma and Paraganglioma | PCPG |
| Prostate adenocarcinoma | PRAD |
| Rectum adenocarcinoma | READ |
| Sarcoma | SARC |
| Skin Cutaneous Melanoma | SKCM |
| Stomach adenocarcinoma | STAD |
| Testicular Germ Cell Tumors | TGCT |
| Thyroid carcinoma | THCA |
| Thymoma | THYM |
| Uterine Corpus Endometrial Carcinoma | UCEC |
| Uterine Carcinosarcoma | UCS |
| Uveal Melanoma | UVM |
